# Systemic and Mucosal Immunogenicity of Monovalent XBB.1.5-Adapted COVID-19 mRNA Vaccines in Patients with Inflammatory Bowel Disease

**DOI:** 10.3390/vaccines12070774

**Published:** 2024-07-15

**Authors:** Simon Woelfel, Joel Dütschler, Daniel Junker, Marius König, Georg Leinenkugel, Nicole Graf, Claudia Krieger, Samuel Truniger, Annett Franke, Seraina Koller, Katline Metzger-Peter, Melanie Oberholzer, Nicola Frei, Nora Geissler, Peter Schaub, STAR SIGN Investigators, Werner C. Albrich, Matthias Friedrich, Jan Hendrik Niess, Nicole Schneiderhan-Marra, Alex Dulovic, Wolfgang Korte, Justus J. Bürgi, Stephan Brand

**Affiliations:** 1 Department of Gastroenterology and Hepatology, Cantonal Hospital St. Gallen, 9007 St. Gallen, Switzerland; 2 Max von Pettenkofer Institute of Hygiene and Medical Microbiology, Faculty of Medicine, Ludwig Maximilian University (LMU), 80333 Munich, Germany; 3 Outpatient Clinic, Ambulatory Services Rorschach, 9400 Rorschach, Switzerland; 4 NMI Natural and Medical Sciences Institute at the University of Tübingen, 72770 Reutlingen, Germany; 5 Department of Gastroenterology and Hepatology, University Digestive Healthcare Center, Clarunis, 4002 Basel, Switzerland; 6 Clinical Trials Unit, Cantonal Hospital St. Gallen, 9007 St. Gallen, Switzerland; 7 Center for Laboratory Medicine, 9001 St. Gallen, Switzerland; 8 Division of Infectious Diseases, Infection Prevention, & Travel Medicine, Cantonal Hospital St. Gallen, 9007 St. Gallen, Switzerland; 9 Translational Gastroenterology Unit, Nuffield Department of Medicine, University of Oxford, Oxford OX3 9DU, UK; 10 Gastroenterology Group, Department of Biomedicine, University of Basel, 4031 Basel, Switzerland

**Keywords:** XBB.1.5-adapted COVID-19 vaccines, mRNA vaccines, inflammatory bowel disease, anti-TNF therapy, SARS-CoV-2, COVID-19, omicron, mucosal immunogenicity, EG.5.1, BA.2.86, STAR SIGN study

## Abstract

Recently updated COVID-19 mRNA vaccines encode the spike protein of the omicron subvariant XBB.1.5 and are recommended for patients with inflammatory bowel disease (IBD) on immunosuppressive treatment. Nonetheless, their immunogenicity in patients with IBD against rapidly expanding virus variants remains unknown. This prospective multicenter cohort study is the first study to investigate the immunogenicity of XBB.1.5-adapted vaccines in patients with IBD. Systemic and mucosal antibodies targeting the receptor-binding domains (RBDs) of the omicron subvariants XBB.1.5, EG.5.1, and BA.2.86, as well as their neutralization were quantified before and two to four weeks after vaccination with monovalent XBB.1.5-adapted mRNA vaccines. Vaccination increased levels of serum anti-RBD IgG targeting XBB.1.5, EG.5.1, and BA.2.86 (1.9-fold, 1.8-fold, and 2.6-fold, respectively) and enhanced corresponding neutralization responses (2.3-fold, 3.1-fold, and 3.5-fold, respectively). Following vaccination, anti-TNF-treated patients had reduced virus neutralization compared to patients on treatments with other cellular targets. 11.1% and 16.7% of patients lacked EG.5.1 and BA.2.86 neutralization, respectively; all these patients received anti-TNF treatment. At mucosal sites, vaccination induced variant-specific anti-RBD IgG but failed to induce RBD-targeting IgA. Our findings provide a basis for future vaccine recommendations while highlighting the importance of frequent booster vaccine adaptation and the need for mucosal vaccination strategies in patients with IBD.

## 1. Introduction

COVID-19 vaccines are crucial pillars for the control of SARS-CoV-2 infections and have prevented millions of deaths since the start of the pandemic in 2020 [1]. Despite the transformation of the pandemic to seasonal outbreaks with epidemic character, vaccines remain an essential tool for combating emerging virus variants that overcome immune responses induced by prior infection or vaccination and protect against post-acute COVID-19 syndrome [2,3]. COVID-19 mRNA vaccines are tailored to induce antibodies against the viral spike protein, which neutralize the virus by preventing binding to its host cell receptor, ACE2 [4]. Immunosuppressive treatment often impairs vaccine-induced immunogenicity and therefore predisposes affected individuals to an elevated burden during ongoing waves of COVID-19 [5]. This includes patients with inflammatory bowel disease (IBD), a widespread multi-factorial autoimmune disorder that leads to chronic gut inflammation and often requires immunosuppressive treatment [6]. Several treatments, including TNF-antagonizing (anti-TNF) therapies, impair the humoral immune response induced by COVID-19 mRNA vaccines after two and three vaccine doses [7,8,9,10]. This leads to reduced levels of virus-targeting antibodies and poor neutralization of wildtype SARS-CoV-2 and several omicron subvariants following vaccination in these patients [11,12,13]. In the general population, reduced antibody responses following COVID-19 vaccination are associated with a higher risk of breakthrough infection [14]. In patients with IBD, breakthrough infections following vaccination are common and more frequent in individuals with poor virus neutralization following vaccination compared to those with robust virus neutralization [10,15,16]. Consequently, anti-TNF-treated patients with IBD face an increased risk of breakthrough infection [11,17]. Collectively, these studies demonstrate an elevated risk for patients with IBD on anti-TNF therapy during ongoing COVID-19 surges, which mandates close monitoring of immune responses in these patients.

To date, high levels of vaccine-mediated immunity against SARS-CoV-2 in the general population exert a high evolutionary pressure on the virus to circumvent existing immune responses induced by vaccination or infection. This resulted in the evolution of the omicron subvariant XBB.1.5 which harbors several spike mutations that facilitate immune evasion [18,19]. An updated generation of mRNA vaccines has been developed to efficiently combat XBB.1.5 and other circulating virus variants. These new vaccines specifically target the spike protein of XBB.1.5 and are recommended for patients with IBD in most countries [20]. However, it remains obscure if XBB.1.5-adapted mRNA vaccines induce sufficient immunogenicity against recent SARS-CoV-2 variants in patients with IBD.

Therefore, this study evaluated systemic and mucosal humoral immune responses induced by monovalent XBB.1.5-adapted mRNA vaccines in patients with IBD and assessed subsequent virus neutralization against immune-evading omicron subvariants.

## 2. Materials and Methods

### 2.1. Study Design, Recruitment, and Procedures

The STAR SIGN (**S**ystemic and **T**-cell-**a**ssociated **r**esponses to **S**ARS-CoV-2 **i**mmunization in **g**ut i**n**flammation) study is conceptualized as a longitudinal multicenter observational cohort study across several Swiss tertiary IBD centers and aims to investigate the immunogenicity of COVID-19 vaccines in patients with IBD. This study was approved by the Ethics Committee of Eastern Switzerland (project-ID 2021-02511). Patients included in this analysis were recruited at the outpatient clinics of the Cantonal Hospital St. Gallen, Ambi Rorschach, and the Digestive Healthcare Center Clarunis, Basel. Included participants were adults with a diagnosis of IBD (ulcerative colitis or Crohn’s disease) on IBD therapy (infliximab, vedolizumab, ustekinumab, or tofacitinib) who had received three doses of original (non-adapted) monovalent COVID-19 mRNA vaccines (BNT162b2 or mRNA-1273) and had no infection or COVID-19 vaccination within six months before inclusion. Patients who received treatment with steroids, immunomodulators (azathioprine, 6-mercaptopurine, or methotrexate), or checkpoint inhibitors (anti-PD-1, anti-PD-L1, or anti-CTLA-4) were excluded from this study. Upon study inclusion, participants were invited to the respective outpatient clinic to fill out a study questionnaire on baseline characteristics and provide serum and saliva samples to evaluate baseline systemic and mucosal immune responses, respectively. After serum collection, participants received a fourth vaccine dose with a monovalent XBB.1.5-adapted COVID-19 mRNA vaccine (BNT162b2 XBB.1.5 or mRNA-1273.815). Two to four weeks later, patients were invited again to the respective outpatient clinic to fill out a study questionnaire on vaccine-induced adverse events and provide serum and saliva samples to evaluate vaccine-induced systemic and mucosal immune responses, respectively.

### 2.2. Serological and Immunochemical Assays

Levels of IgG and IgA targeting the receptor binding domain (RBD) of the SARS-CoV-2 omicron subvariants XBB.1.5, EG.5.1, and BA.2.86 were assessed using MULTICOV-AB, a multiplex immunoassay allowing for the paralleled quantification of antibodies targeting different antigens [21]. Anti-RBD IgG levels were quantified in serum and saliva samples, while anti-RBD IgA levels were quantified in saliva samples exclusively. The assay procedure was described previously [22]. In brief, samples were diluted in assay buffer, and 25 µL was mixed at a 1:1 ratio with a pool of variant-specific RBDs, which were immobilized on spectrally distinct beads. Sample RBD-bead conjugate mixes were incubated for 2 h at 20 °C with 750 rpm agitation in a 96-well plate (Corning, Corning, NY, USA). After removing unbound serum antibodies by washing three times with a microplate washer (Biotek 405TS, Biotek Instruments GmbH, Bad Friedrichshall, Germany), samples were incubated with 3 µg/mL RPE-huIgG (Dianova, Hamburg, Germany) or 5 µg/mL RPE-huIgA (Dianova) for 45 min at 20 °C with 750 rpm agitation, followed by an additional wash step. Beads were resuspended in 100 µL wash buffer and incubated for 3 min at 20 °C with 1000 rpm agitation. Bound antibodies were quantified by determining the mean fluorescence intensity (MFI) using a FLEXMAP 3D instrument (Luminex, Austin, TX, USA). Raw MFI values were normalized to cutoff samples with a known MFI.

Serum-mediated neutralization of omicron subvariants XBB.1.5, EG.5.1, and BA.2.86 was determined through RBDCoV-ACE2 multiplex immunoassay as described previously [13]. This assay employs the inhibition of binding between the virus RBD and its receptor, ACE2, by neutralizing antibodies as a robust surrogate. This approach was validated previously and has been used in many studies assessing COVID-19 mRNA vaccine immunogenicity [23,24,25,26,27,28]. Briefly, each subvariant specific RBD protein was coupled to a single type of MagPlex beads (Luminex) with unique spectral properties. Beads were pooled to reach a final concentration of 40 beads/µL for each bead-RBD couple. Serum samples were diluted in assay buffer and mixed with biotinylated human ACE2 protein (Sino Biological, Beijing, China) to a final concentration of 300 ng/µL, and 25 µL of diluted serum was mixed with bead mix in a 1:1 ratio using a 96-well plate (Corning). Samples were incubated for 2 h at 21 °C and 750 rpm agitation, and unbound proteins were removed by washing three times using a microplate washer (Biotek Instruments GmbH). Next, 30 µL of streptavidin-RPE conjugate was added, and the plate was incubated for 45 min at 21 °C and 750 rpm agitation. Following three more wash cycles, the plate was incubated for 3 min at 1000 rpm before quantification. Bead-attached biotinylated ACE2 protein was quantified by determining the MFI of conjugated RPE using a FLEXMAP 3D instrument (Luminex). MFI values of each sample well were divided by the mean of normalization controls that did not contain any serum. Resulting values were converted into percentage and subtracted from 100 to obtain the percentage of ACE2 binding inhibition. Serum samples with ACE2 binding inhibition of less than 20% were considered non-neutralizing, as validated previously [24].

### 2.3. Study Outcomes

Primary outcomes were levels of IgG targeting the RBDs of the omicron subvariants XBB.1.5, EG.5.1, and BA.2.86 in sera before and two to four weeks after fourth-dose vaccination with XBB.1.5-adapted COVID-19 mRNA vaccines, and the corresponding serum-mediated neutralization against these subvariants.

Secondary outcomes:

• Mucosal vaccine immunogenicity, as determined by levels of IgG and IgA targeting the RBDs of the omicron subvariants XBB.1.5, EG.5.1, and BA.2.86 in saliva before and two to four weeks after vaccination.
• Vaccination-induced adverse events within seven days after vaccination.

### 2.4. Statistical Analysis

In Table 1, categorical variables are shown with absolute and relative frequencies, and continuous variables are summarized with mean and standard deviation (SD). Statistical analyses were performed using R version 4.2.2, and figures were plotted with GraphPad Prism version 9.3.1. The exact Wilcoxon signed-rank test was used to compare dependent samples, such as anti-RBD IgG and IgA levels, and serum-mediated neutralization before and after vaccination. The exact Wilcoxon rank sum test was used to compare independent samples, such as anti-RBD IgG and IgA levels, and serum-mediated neutralization between patients on anti-TNF therapy and those on non-anti-TNF therapy. Bivariate correlation analyses between variables were performed by calculating Spearman’s rho and corresponding *p* values.

## 3. Results

### 3.1. Study Population

Between November 2023 and February 2024, 290 patients with IBD were contacted and screened for study eligibility at two Swiss tertiary IBD centers. For various reasons, only 18 patients who fulfilled all inclusion criteria consented to study participation and were enrolled in this substudy of the STAR SIGN study, focusing on the immunogenicity of XBB.1.5-adapted mRNA vaccines (Figure S1). The mean age of participants was 49.3 (SD 16.8) years, 44.4% were female, and 55.6% were male (Table 1). In total, 11 of 18 participants (61.1%) received infliximab as IBD therapy, 5 (27.8%) received vedolizumab, 1 (5.6%) received ustekinumab, and 1 (5.6%) was treated with tofacitinib. Study population characteristics stratified by the cellular targets of IBD therapy (anti-TNF or non-anti-TNF) are listed in Table S1. Subsequently, patients were subjected to fourth-dose vaccination with XBB.1.5-adapted COVID-19 mRNA vaccines. In total, 16 of 18 participants (88.9%) received BNT162b2 XBB.1.5 and 2 (11.1%) received mRNA-1273.815. Across the study population, eleven individuals (61.1%) experienced local adverse events, and eight individuals (44.4%) experienced systemic adverse events in response to vaccination with XBB.1.5-adapted vaccines (Table S2).

### 3.2. XBB.1.5-Adapted COVID-19 Vaccines Induce Systemic Humoral and Neutralizing Immunity against Omicron Subvariants in Patients with IBD

**Figure 1:**
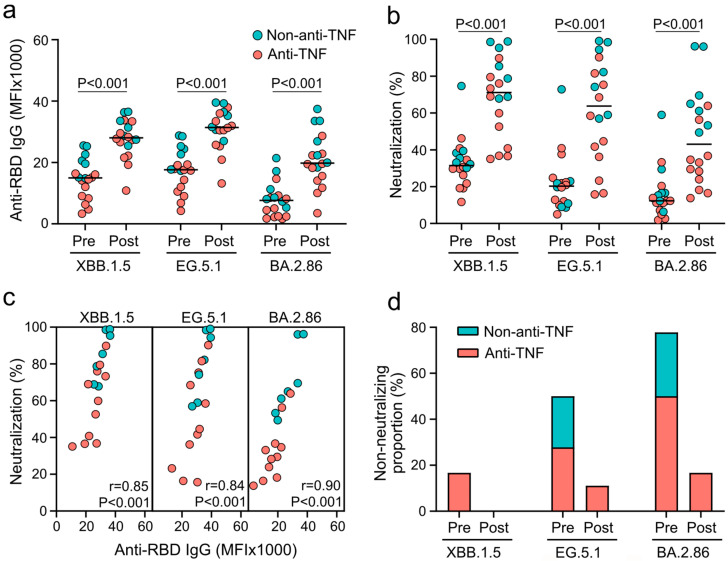
Systemic antibody responses and corresponding virus neutralization induced by XBB.1.5-adapted mRNA vaccines in patients with IBD. (**a**) Median serum levels of omicron subvariant-specific anti-RBD IgG before (pre) and 2–4 weeks after (post) vaccination. (**b**) Median serum-mediated neutralization of indicated omicron subvariants before and after vaccination. Neutralization is based on antibody-mediated inhibition of binding between ACE2 and the indicated RBDs. (**c**) Bivariate correlation between subvariant-specific anti-RBD IgG levels and respective virus neutralization 2–4 weeks after vaccination. Correlation coefficients are provided as Spearman’s rho and corresponding *p* values. (**d**) Proportions of individuals who fail to neutralize the indicated subvariants before and after vaccination. Statistical analyses in (**a**,**b**) are based on exact Wilcoxon signed-rank tests. Color code: anti-TNF-treated patients are depicted in red, and non-anti-TNF-treated patients are displayed in blue.

Vaccination with XBB.1.5-adapted mRNA vaccines increased the serum levels of IgG targeting the RBD of the omicron subvariants XBB.1.5, EG.5.1, and BA.2.86 from medians of 14944 MFI, 17640 MFI, and 7648 MFI to 28022 MFI, 31404 MFI, and 19772 MFI, respectively (each *p* < 0.001; Figure 1a). The most robust vaccine-mediated increase was observed for anti-RBD IgG targeting BA.2.86 (2.6-fold), followed by IgG targeting XBB.1.5 (1.9-fold) and EG.5.1 (1.8-fold). Since previous studies showed a reduction in vaccine-induced immune responses in patients on anti-TNF therapy compared to those on therapy with other cellular targets (non-anti-TNF), we stratified antibody responses by IBD therapy [7,10]. Following vaccination, anti-TNF-treated patients had reduced levels of anti-RBD IgG targeting BA.2.86 compared to non-anti-TNF-treated patients (*p* = 0.012; Figure S2a). These findings suggest that XBB.1.5-adapted vaccines induce systemic humoral immunity against SARS-CoV-2 in patients with IBD in a variant- and treatment-dependent manner.

To assess the functional consequences of vaccine-induced humoral responses, inhibition of RBD-ACE2 receptor binding by neutralizing antibodies was determined as a surrogate for virus neutralization. As validated previously, substantial virus neutralization is indicated by a high percentage of ACE2 binding inhibition in this assay [24]. Vaccination increased virus neutralization from a median of 31.4% (XBB.1.5), 20.3% (EG.5.1), and 12.3% (BA.2.86) to 71.1%, 63.7%, and 43.1%, respectively (each *p* < 0.001; Figure 1b). The highest neutralization increase was observed for BA.2.86 (3.5-fold), followed by EG.5.1 (3.1-fold) and XBB.1.5 (2.3-fold). Patients on anti-TNF therapy showed reduced neutralization of each subvariant following vaccination compared to patients on non-anti-TNF therapy (XBB.1.5: *p* = 0.020, EG.5.1: *p* = 0.020, BA.2.86: *p* = 0.001; Figure S2b). These results indicate that the increase of anti-RBD IgG elicited by XBB.1.5-adapted vaccines translates into increased neutralization against omicron subvariants and is impaired by anti-TNF therapy in patients with IBD. A strong positive correlation between anti-RBD IgG levels after vaccination and corresponding virus neutralization was observed for XBB.1.5, EG.5.1, and BA.2.86 (each *p* < 0.001; Figure 1c). Correlation coefficients were 0.85, 0.84, and 0.90 for XBB.1.5, EG.5.1, and BA.2.86, respectively, indicating that these subvariants possess limited ability to evade vaccine-induced antibodies.

We previously validated that serum samples with an ACE2 binding inhibition of less than 20% fail to neutralize SARS-CoV-2 [24]. Therefore, we assessed the proportions of individuals without neutralization against the tested omicron subvariants in our study cohort. Before vaccination, 16.7% of patients (all anti-TNF recipients) failed to neutralize XBB.1.5, but all patients were XBB.1.5 neutralization competent after vaccination (Figure 1d). Regarding EG.5.1 and BA.2.86, vaccination decreased the proportions of non-neutralizing individuals from 50.0% and 77.8% to 11.1% and 16.7%, respectively. Of note, all patients without neutralization of EG.5.1 and BA.2.86 following vaccination were anti-TNF recipients. Taken together, these results indicate that anti-TNF-treated patients with IBD may be at elevated risk for infections with EG.5.1, BA.2.86, and related omicron subvariants following vaccination despite some risk reduction.

### 3.3. XBB.1.5-Adapted COVID-19 Vaccines Fail to Induce Mucosal IgA Responses against Omicron Subvariants in Patients with IBD

**Figure 2:**
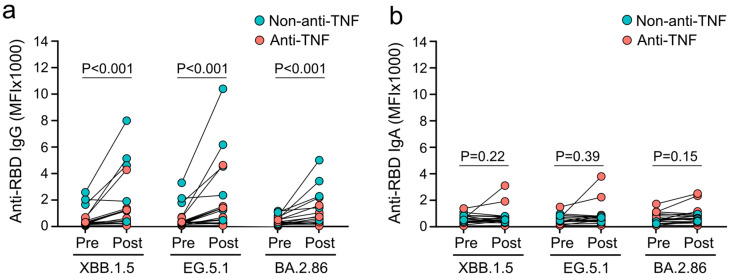
Mucosal antibody responses induced by XBB.1.5-adapted mRNA vaccines in patients with IBD. Omicron subvariant-specific anti-RBD IgG (**a**) and IgA (**b**) levels in saliva, before (pre) and 2–4 weeks after (post) vaccination. Statistical analyses are based on exact Wilcoxon signed-rank tests.

Levels of mucosal IgG targeting the RBD of XBB.1.5, EG.5.1, and BA.2.86 in the saliva of patients with IBD were increased by vaccination with XBB.1.5-adapted vaccines from medians of 302 MFI, 303 MFI, and 326 MFI to 1212 MFI, 1320 MFI, and 674 MFI, respectively (each *p* < 0.001; Figure 2a). In contrast, levels of mucosal IgA targeting the RBDs of XBB.1.5, EG.5.1, and BA.2.86 were not significantly elevated after vaccination compared to pre-vaccination (each *p* ≥ 0.15) and were at low levels following vaccination (XBB.1.5: 538 MFI, EG.5.1: 592 MFI, BA.2.86: 646 MFI; Figure 2b). No significant differences in mucosal IgG or IgA levels were found in patients on anti-TNF therapy compared to patients on non-anti-TNF therapy (Figure S3a,b). Levels of systemic anti-RBD antibodies positively correlated with mucosal anti-RBD antibodies targeting the same subvariant, independent of antibody class (IgG and IgA; Figure S4a,b). These results demonstrate that XBB.1.5-adapted mRNA vaccines elicit mucosal IgG but not mucosal IgA responses in patients with IBD.

## 4. Discussion

Our findings demonstrate that monovalent XBB.1.5-adapted mRNA vaccines elicit serum antibodies that target the RBDs of XBB.1.5, EG.5.1, and BA.2.86 and mediate increased neutralization of these subvariants. While this aligns with data from healthy individuals from other studies, our study is the first to investigate vaccine immunogenicity in patients with inflammatory diseases and immunomodulatory medication [29,30]. We previously reported that third-dose vaccination with original monovalent mRNA vaccines induces poor serum-mediated XBB.1.5 neutralization in patients with IBD, which is further aggravated by anti-TNF therapy [13]. Similarly, patients with anti-TNF therapy had reduced virus neutralization following XBB.1.5-adapted vaccines in the current study, with some patients lacking sufficient neutralization capacity. Provine et al. previously showed that anti-TNF treatment impairs the activation of mucosal-associated invariant T cells using SARS-CoV-2 adenoviral vector vaccines, which in turn reduces the CD8+ T cell immunity to vaccine antigens [31]. Whether the anti-TNF-mediated impairment of neutralizing immune responses induced by mRNA vaccines underlies similar mechanisms remains to be determined. Neutralizing antibodies are critical players in clearing SARS-CoV-2 infections and preventing viral persistence [32]. The lack of neutralizing antibodies in immunocompromised patients may therefore bear the risk of long-term infections, which can facilitate viral adaptation and aid the development of new variants with unknown potential [33]. Accordingly, our data support the frequent application of variant-adapted booster vaccines in anti-TNF therapy recipients to optimize their protection and prevent the evolution of new variants. Our data suggest that XBB.1.5-adapted COVID-19 vaccines are also efficient against closely related omicron subvariants, such as EG.5.1 and BA.2.86, likely due to overlapping antigenic landscapes of their spike proteins. The World Health Organization released a statement on the antigen composition of COVID-19 vaccines in April 2024, in which the adaptation of future COVID-19 vaccines to the spike protein of JN.1, a closely related subvariant of BA.2.86, is recommended [34].

Our results also show that XBB.1.5-adapted vaccines induce mucosal anti-RBD IgG but not IgA in patients with IBD. This is in line with a previous study that detected only modest and highly variable IgA induction by COVID-19 mRNA vaccines in the saliva of healthy individuals [35]. Future studies should aim to reproduce our findings in nasal washes and bronchoalveolar lavage fluids, which may represent the immune response at viral entry sites better than saliva. Emerging SARS-CoV-2 omicron subvariants, including those investigated in this study, can efficiently overcome immune responses triggered by previous infections or vaccinations. Therefore, original vaccines hardly prevent the transmission of such viruses and merely protect from severe disease [36,37]. Mucosal IgA is strongly associated with viral control upon exposure, suggesting that nasally administered vaccines that can trigger robust mucosal IgA responses may effectively prevent virus transmissions [38]. Our data indicate that patients with IBD could benefit from receiving such COVID-19 vaccines to increase protection from SARS-CoV-2 infection and associated short- and long-term ailments. Mucosal vaccines may also require frequent adaptation to circulating variants to maintain high virus protection. A recent study characterized COVID-19 vaccine-elicited IgG responses in patients with celiac disease [39]. Given the pronounced similarities of pro-inflammatory mucosal immune profiles regarding the IgA compartment between patients with IBD and celiac disease, future studies should aim to investigate if XBB.1.5-adapted COVID-19 mRNA vaccines induce similarly weak mucosal IgA responses in patients with celiac disease as seen for patients with IBD in this study [40,41]. When antibodies wane over time or virus variants evolve to escape vaccine-induced antibodies, respiratory-tract-resident CD8^+^ T cells are essential in controlling viral replication in the lung [42]. More research is required to understand the induction of cellular immunity by XBB.1.5-adapted vaccines in patients with IBD. One limitation of this study is the small sample size, which is related to the very difficult study recruitment. Out of 290 invited patients, only 18 fulfilled all inclusion criteria in this substudy of the STAR SIGN study. We cannot rule out that some of our conclusions may be impacted by this limitation and advice that our findings should be confirmed in larger studies. The neutralization assay used in this study is based on the binding inhibition of the SARS-CoV-2 RBD to its host receptor ACE2. This intrinsically implicates limitations compared to live SARS-CoV-2 neutralization assays. However, given the robust data from previous studies using this assay combined with the strong correlation between anti-RBD IgG and neutralization after vaccination observed in this study, we assume that the assay performance is sufficient to support the conclusions drawn from the presented data [22,24,26]. While the number of previous infections, the proportions of underlying diseases, and the time between fourth-dose vaccination and sampling were comparable between patients on anti-TNF and non-anti-TNF therapy in our study, we cannot rule out that our comparisons between these groups are impacted by the type of variant an individual was previously infected with.

In summary, this study provides valuable insights into the efficacy of XBB.1.5-adapted vaccines in patients with IBD. It highlights the importance of continuous vaccination campaigns to revamp immunity towards emerging SARS-CoV-2 variants and continue to succeed in the molecular arms race against an ever-evolving pathogen.

## Figures and Tables

**Table 1:** Study population baseline characteristics.

	Study Population (n = 18)
Age, years (SD)	49.3 (16.8)
Sex (%)	
Female	8 (44.4)
Male	10 (55.6)
Other	0 (0.0)
BMI, kg/m^2^ (SD)	21.9 (4.3)
Ethnicity (%)	
European	17 (94.4)
Asian	0 (0.0)
African	0 (0.0)
Others	1 (5.6)
Smoking status (%)	
Never	8 (44.4)
Former	7 (38.9)
Current	3 (16.7)
Diagnosis (%)	
Ulcerative colitis	10 (55.6)
Crohn’s disease	7 (38.9)
Indeterminate colitis	1 (5.6)
Duration of IBD, years (SD)	15.3 (10.8)
PRO2-based disease activity ^1^ (%)	3 (16.7)
Fecal calprotectin-based disease activity ^2^ (%)	8 (44.4)
IBD therapy (%)	
Infliximab (anti-TNF)	11 (61.1)
Adalimumab (anti-TNF)	0 (0.0)
Certolizumab pegol (anti-TNF)	0 (0.0)
Golimumab (anti-TNF)	0 (0.0)
Vedolizumab (non-anti-TNF)	5 (27.8)
Ustekinumab (non-anti-TNF)	1 (5.6)
Tofacitinib (non-anti-TNF)	1 (5.6)
Systemic steroids	0 (0.0)
Immunomodulators	0 (0.0)
Underlying disease (%)	
Cancer	2 (11.1)
Heart disease	2 (11.1)
Hypertension	3 (16.7)
Pulmonary disease	2 (11.1)
Kidney disease	3 (16.7)
Diabetes	0 (0.0)
Arthritis	3 (16.7)
Hyperlipidemia	1 (5.6)
Liver disease	2 (11.1)
SARS-CoV-2 infection since third vaccination (%)	6 (33.3)
Number of SARS-CoV-2 infections ever (%)	
0	8 (44.4)
1	9 (50.0)
2	0 (0.0)
3	1 (5.6)
Type of fourth-dose vaccine	
BNT162b2 XBB.1.5	16 (88.9)
mRNA-1273.815	2 (11.1)
Vaccination schedule doses 1–4 (%)	
Homologous	15 (83.3)
Heterologous	3 (16.7)

^1^ PRO2 constitutes a disease activity score based on patient-reported outcomes. ^2^ Disease is considered active if fecal calprotectin concentration is above a threshold of 50 µg/g.

## Data Availability

Data underlying the presented study will be made available as anonymized raw data upon reasonable request to the corresponding author. Additional analyses of study data must be in line with the approved study protocol. Data transfer requires written consent to a transfer agreement before transfer.

## Supplementary Materials

The following supporting information can be downloaded at: https://www.mdpi.com/article/10.3390/vaccines12070774/s1, Figure S1: Study recruitment scheme; Figure S2: Systemic antibody responses and corresponding virus neutralization following immunization with XBB.1.5-adapted vaccines, stratified by IBD therapy; Figure S3: Mucosal antibody responses following immunization with XBB.1.5-adapted vaccines, stratified by IBD therapy; Figure S4: Correlation of serum and saliva IgG and IgA levels following immunization with XBB.1.5-adapted vaccines; Table S1: Study population characteristics stratified by treatment group; Table S2: Adverse events in response to XBB.1.5-adapted COVID-19 mRNA vaccines; Table S3: STAR SIGN study investigators.
