# Supplementary material for: Systemic and Mucosal Immunogenicity of Monovalent XBB.1.5-Adapted COVID-19 mRNA Vaccines in Patients with Inflammatory Bowel Disease
Source: Vaccines (Basel). 2024 Jul 15;12(7):774. doi: 10.3390/vaccines12070774 (PMC11281571; doi:10.3390/vaccines12070774)
Supplement: Supplementary file 1 [file vaccines-12-00774-s001.zip › vaccines-3073185-supplementary.pdf]

vaccines

Supplementary Materials

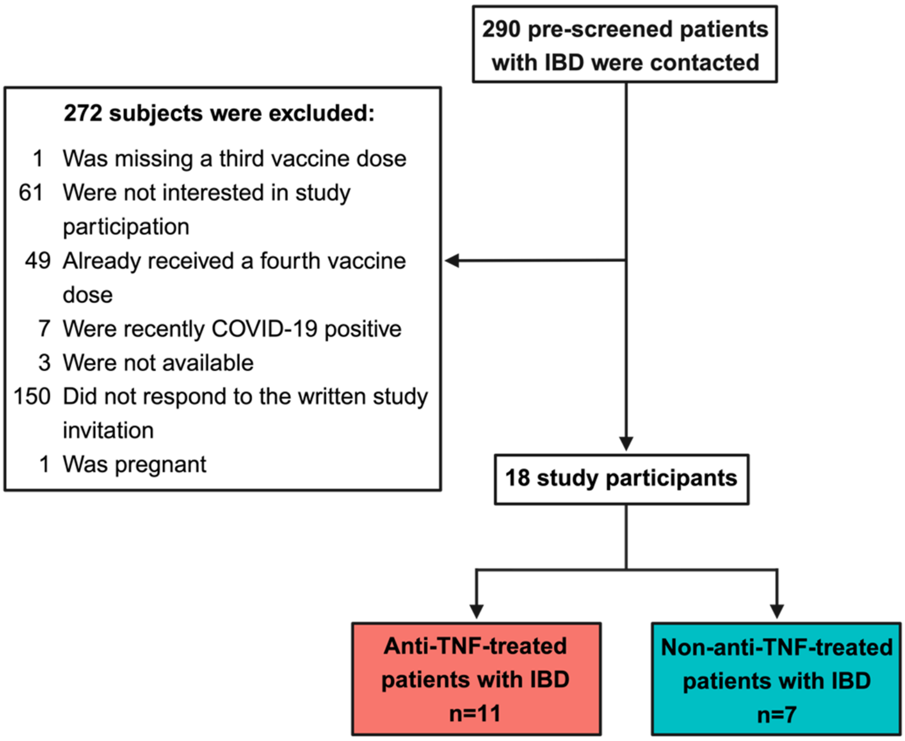

Figure S1. Study recruitment scheme. All participants received a fourth vaccine dose with XBB.1.5-adapted COVID-19 mRNA vaccines.

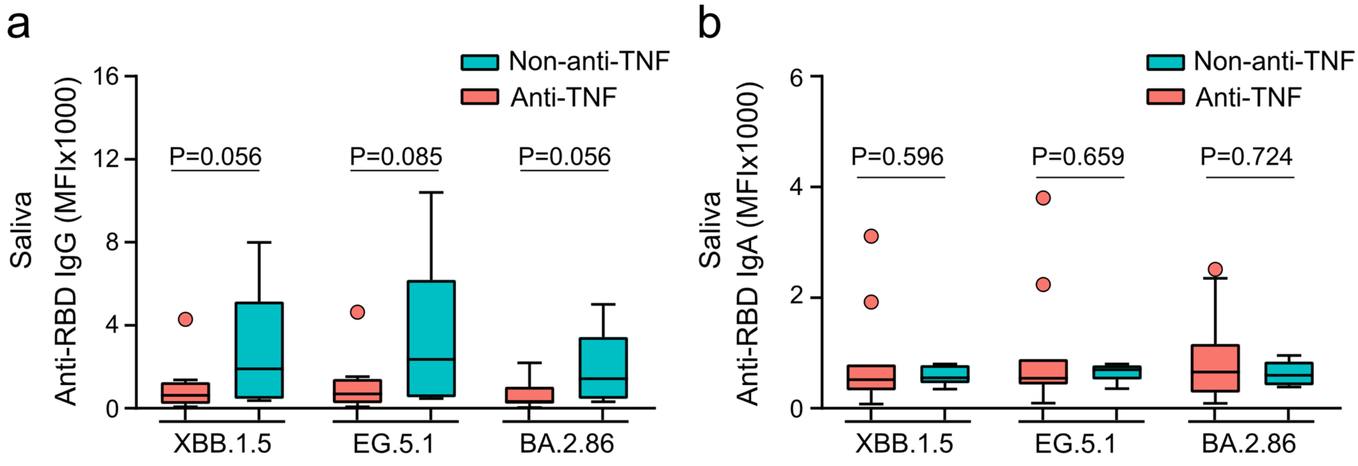

Figure S2. Systemic antibody responses and corresponding virus neutralization following immunization with XBB.1.5-adapted vaccines, stratified by IBD therapy. (**a**) Serum levels of omicron subvariant-specific anti-RBD IgG, 2-4 weeks after vaccination, stratified by IBD treatment. Boxes depict median and interquartile range (IQR), top whiskers indicate the largest value within 75th percentile plus 1.5x IQR, bottom whiskers indicate the lowest value within 25th percentile minus 1.5x IQR. (**b**) Serum-mediated neutralization of indicated omicron subvariants, 2-4 weeks after vaccination, stratified by IBD treatment. Neutralization is based on antibody-mediated inhibition of binding between ACE2 and the indicated RBDs. Boxes depict median and IQR, whiskers indicate last value within 1.5x IQR. Statistical analyses are based on exact Mann Whitney tests.

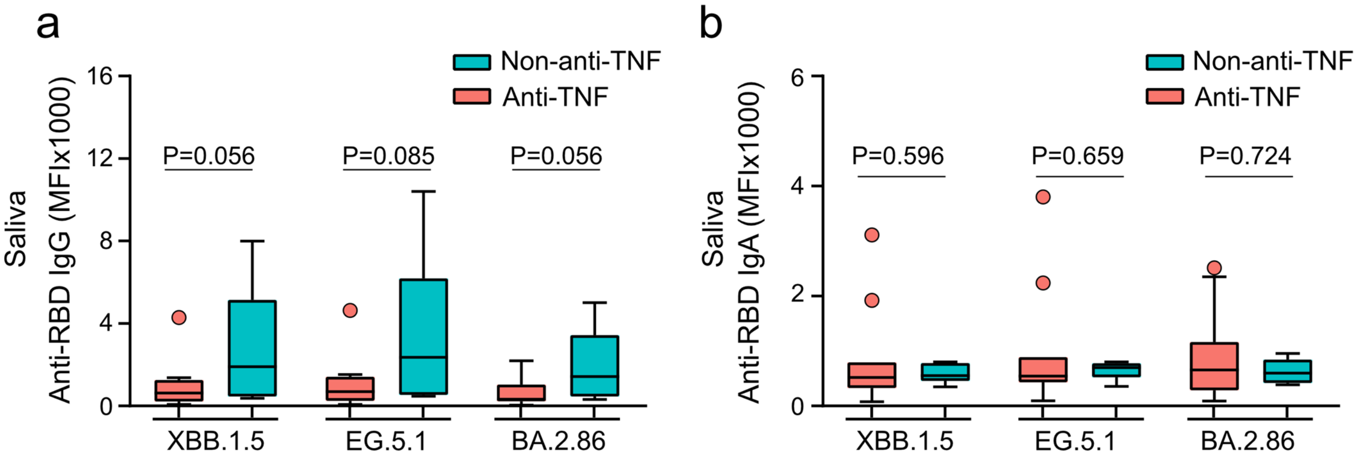

Figure S3. Mucosal antibody responses following immunization with XBB.1.5-adapted vaccines, stratified by IBD therapy. (**a**) and (**b**) Levels of omicron subvariant-specific anti-RBD IgG (**a**) and IgA (**b**) in saliva 2-4 weeks after vaccination, stratified by IBD treatment. Boxes depict median and interquartile range (IQR), top whiskers indicate the largest value within 75th percentile plus 1.5x IQR, bottom whiskers indicate the lowest value within 25th percentile minus 1.5x IQR. Values outside of the whisker ranges are depicted as dots. Statistical analyses are based on exact Mann Whitney tests.

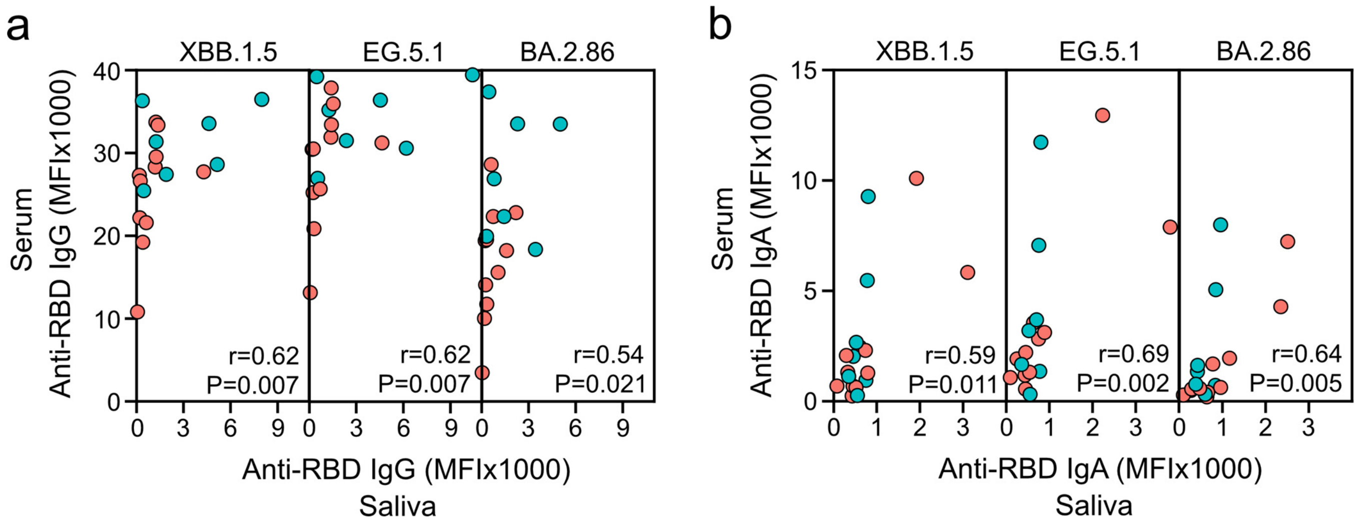

Figure S4. Correlation of serum and saliva IgG and IgA levels following immunization with XBB.1.5-adapted vaccines. Depicted are bivariate correlations of values 2-4 weeks after vaccination with XBB.1.5-adapted vaccines. Correlation coefficients are provided as Spearman's rho and corresponding P value. Color-code: anti-TNF-treated patients are depicted in red, non-anti-TNF-treated patients are depicted in blue.

Table S1. Study population characteristics stratified by treatment group.

	**Anti-TNF (n=11)**	**Non-anti-TNF (n=7)**
Number of previous SARS-CoV-2 infections [1] (%)		
0	5 (45.5)	3 (42.9)
1	5 (45.5)	4 (57.1)
2	0 (0.0)	0 (0.0)
3	1 (9.1)	0 (0.0)
Time span between vaccination and sample collection in days, mean [2] (SD)	20.2 (4.8)	18.0 (4.7)
Underlying disease [1] (%)		
Cancer	1 (9.1)	1 (14.3)
Heart disease	1 (9.1)	1 (14.3)
Hypertension	1 (9.1)	1 (14.3)
Pulmonary disease	1 (9.1)	1 (14.3)
Kidney disease	1 (9.1)	2 (28.6)
Diabetes	0 (0.0)	0 (0.0)
Arthritis	3 (27.3)	0 (0.0)
Hyperlipidemia	1 (9.1)	0 (0.0)
Liver disease	2 (18.2)	0 (0.0)

[1] None of the differences in percentages were significant using two-tailed Fisher's exact tests (each $p>0.05$).
[2] The difference was not significant using Student's t test ($p>0.05$).

Table S2. Adverse events in response to XBB.1.5-adapted COVID-19 mRNA vaccines.

	**Study population (n=18)**
Local adverse events (%)	
Any	11 (61.1)
Pain	11 (61.1)
Erythema	2 (11.1)
Local swelling	2 (11.1)
Axillary swelling	1 (5.6)
Systemic adverse events (%)	
Any	8 (44.4)
Fever	3 (16.7)
Headache	4 (22.2)
Fatigue	4 (22.2)
Myalgia	3 (16.7)
Arthralgia	1 (5.6)
Nausea	1 (5.6)
Chills	1 (5.6)

Table S3: STAR SIGN study investigators.

**First name**	**Last name**
Benjamin	Misselwitz
Vasileios	Oikonomou
Jacqueline	Wyss
Niklas	Krupka
Irina	Bergamin
Reto	Bertolini
Jan	Borovicka
Remus	Frei
Johannes	Haarer
Rahel	Häuptle
Pamela	Meyer-Herbon
Sandra	Müller
Mikael	Sawatzki
Alexandra	Schwizer
Gian-Marco	Semadeni
David	Semela
Sarah	Zwyssig
